# Comparison of analgesic effects between erector spinae and transversus abdominis plane blocks in patients undergoing laparoscopic cholecystectomy

**DOI:** 10.12669/pjms.40.3.8284

**Published:** 2024

**Authors:** Hui Chen, Junshi Li, Jianfeng Zuo, Xiping Zhang

**Affiliations:** 1 Hui Chen, Department of Anesthesiology, Changxing County People’s Hospital, Changxing Country, Huzhou 313100, Zhejiang Province, P.R. China; 2 Junshi Li, Department of Anesthesiology, Changxing County People’s Hospital, Changxing Country, Huzhou 313100, Zhejiang Province, P.R. China; 3 Jianfeng Zuo, Department of Anesthesiology, Changxing County People’s Hospital, Changxing Country, Huzhou 313100, Zhejiang Province, P.R. China; 4 Xiping Zhang, Department of Anesthesiology, Changxing County People’s Hospital, Changxing Country, Huzhou 313100, Zhejiang Province, P.R. China

**Keywords:** Analgesic effect, Block, Erector spine plane, Laparoscopic cholecystectomy, Transversus abdominis plane

## Abstract

**Objective:**

To compare the analgesic effects and incidence of postoperative adverse events after the erector spine plane (ESP) and transversus abdominis plane (TAP) blocks in patients undergoing laparoscopic cholecystectomy (LC).

**Methods:**

In this retrospective observational study, clinical data of 103 patients undergoing LC in Changxing County People’s Hospital from October 2020 to October 2022 were retrospectively reviewed, and the patients were divided into ESP-group (n=56) and TAP-group (n=57) based on the block method. The operation time, the change of visual analogue scale (VAS) score of static (sVAS) and dynamic (dVAS) pain after operation, the patient-controlled dose, and the remedial analgesic dose at 24 hours after the operation were compared between the two groups. The occurrence of postoperative adverse reactions in both groups was recorded.

**Results:**

The dVAS scores of the ESP-group at one hour, three hours, six hours, and 12 hours after the operation were lower than those of the TAP-group (*P*<0.05). The patient-controlled dose and remedial analgesia dose of the ESP-group were significantly lower than those of the TAP-group (*P*<0.05). There was no difference in the incidence of postoperative nausea and vomiting between the two groups (*P*>0.05).

**Conclusions:**

ESP block and TAP block in LC patients have similar operation time and incidence of postoperative adverse events such as nausea and vomiting. However, short-term postoperative analgesic effect of ESP block is superior to TAP and requires a lower dose of analgesia.

## INTRODUCTION

Laparoscopic cholecystectomy (LC) is a minimally invasive operation that has become the clinical gold standard for daytime gallbladder surgery.^1^,^2^ However, LC is associated with significant pain during the perioperative period, which is mainly related to intraoperative traction reaction, abdominal wall incision pain, and hypercapnia.^3^ Opioid drugs and non-steroidal analgesics have been widely used for clinical analgesia in the past. However, these drugs can be associated with opioid dependence, excessive sedation, respiratory depression, and other adverse reactions.^4^ With the continuous development of ultrasound-guided regional nerve block, peripheral nerve block technology is gradually applied to perioperative analgesia.^5^

Transversus abdominis plane (TAP) covers T6 to L1 sensory nerves. Local anesthetic injection at this plane can block the sensory nerve and exert postoperative analgesic effect.^6^ In 2001, Rafi proposed TAP block anesthesia for postoperative analgesia after laparoscopic surgery.^6^,^7^ Subsequently, this technology became widely used in postoperative analgesia of different types of laparoscopic surgery, such as laparoscopic colectomy and hernia repair.^7^ Erector spine plane (ESP) block is a regional nerve block method that inhibits and blocks the conduction of dorsal or ventral branches of deep spinal nerve by injecting local anesthetic drugs into the deep side of the fascia of erector spinal muscle.^8^ Research reports that both ultrasound-guided ESP block and TAP block can be used to relieve pain during upper abdominal surgery.^9^

However, there are few comparative studies on the application of the two methods in LC, and there is still no consensus on the efficiency of these methods in postoperative analgesia and the rate of postoperative nausea and vomiting. The current retrospective study reviewed clinical data of 103 LC patients from October 2020 to October 2022 to compare the analgesic effect of ESP and TAP block, and investigate their impact on postoperative adverse events.

## METHODS

In this retrospective observational study, a total of 103 patients (50 males and 63 females) who underwent LC in Changxing County People’s Hospital from October 2020 to October 2022 were selected.SSS The mean (SD) age of the patients was 51.82 (11.72) years. According to the surgical block records of the patients, 56 of them received ESP block were set as ESP-group, and 57 patients received TAP block were set as TAP-group.

### Inclusion criteria:

- The American Society of Anesthesiologists (ASA) Grade-I~II.
- The New York Heart Association (NYHA) classification: Grade-I and II.
- Age ≥ 18 years old.
- Patients without history of abdominal surgery
- The clinical data was complete.

### Exclusion criteria:

- Patients with coagulation dysfunction.
- Patients with peripheral neuropathy.
- Patients with abnormal liver and kidney function.
- Patients with severe cardiovascular disease.
- Patients who were allergic to the anesthetic drugs used in this study.
- Complicated with chronic bronchitis.
- Patients with chronic pain.

### Ethical Approval:

This study was reviewed and approved by the Changxing County People’s Hospital ethics committee (Number: 20230411, Date: 2023-04-21).

All patients underwent routine monitoring of electrocardiogram (ECG), blood pressure, heart rate and blood oxygen saturation. Before operation, venous access was established. Midazolam (Jiangsu Nhwa Pharmaceutical Co., Ltd., Approval No. H19990027) 0.02mg/kg and sufentanil (Yichang Renfu Pharmaceutical Co., Ltd., Approval No. H20054171) 0.01μg/kg were administered intravenously for sedation. Nerve block was done by the anesthesiologist 30 minutes before the anesthesia induction.

### ESP block:^10^

The patient was placed in a lateral position, and received routine disinfection and tissue laying. A 15MHz high-frequency linear array ultrasonic probe was then used to scan the spine in the midsagittal position, count down to the horizontal along the horizontal direction of the C7 spinous process, determine the location of the T7 spinous process, slide from the middle to both sides to form a parallel view, from the outside to the inside. The sequence was skin, subcutaneous tissue, trapezius muscle and then the vertical spine muscle. At the lower edge of the rhomboid muscle, T5 vertebral body and T6 vertebral body level, the position of T7 transverse process can be determined by observing the missing part of this layer. The probe was rotated 90°, and the superficial tissue was infiltrated with local anesthesia. Under the guidance of ultrasound and the in-plane technology, the puncture needle (22G 100mm) was inserted in the direction of the tail, and the angle was controlled in the range of 30°~40°.

The needle penetrated the deep surface of the fascia of the vertical spine muscle, until the T7 transverse process. After contacting it, 3mL~5mL of normal saline was injected, and the condition of the vertical spine muscle were observed to make sure it is floating without swelling. Then, 20mL 0.3% ropivacaine (Jiangsu Hengrui Pharmaceutical Co., Ltd., Approval number H20060137) was injected. The probe moved along the direction of the head and tailbone of the patient, and the diffusion direction of the injected drug was confirmed. The separation of the vertical spine muscle from the transverse was observed. The same method was used for the opposite side to block the nerve. After 20 minutes, the blocking plane was determined using the ice method.

### TAP block:^11^

TAP block was performed using the subcostal approach. Probe was placed on the upper abdominal wall of the patient in the supine position, and gradually moved closer to the xiphoid direction near the midline of the abdomen along the lower edge of the rib. The probe continued to move laterally until the aponeurosis of the external oblique muscle was reached, and the internal oblique muscle and the transverse abdominal fascia were visible. Then the probe was moved horizontally until the transverse abdominal muscle became visible. The anesthesiologist pointed the needle at the transverse abdominal fascia and injected 20 mL of 0.3% ropivacaine into the rectus abdominis and through the abdominal muscles along the subcostal line. The above steps were then repeated for the opposite side. The detection method of tissue plane exploration was the same as that of ESP.

All patients were given general anesthesia and underwent LC. After operation, the visual analogue scale (VAS) score of patients was assessed. VAS is a measure for pain intensity. It consists of a 10cm line, with both ends representing 0 (’no pain’) and 10 (’worst imaginable pain’).^12^ In cases of score ≥ 5 points, flurbiprofen axetil (Beijing Tide Pharmaceutical Co., Ltd., Approval No.: H20041508) 50mg was given by intravenous injection for remedial analgesia. Within 48 hours after operation, the postoperative VAS score of all patients was less than five points. Basic data of the patients were collected and the VAS scores of static and dynamic were collected at one, three, six, 12, and 24 hours after operation.

The patient-controlled analgesia (PCA) dose and the measurement of relief analgesia were calculated after operation. PCA preparation contained 100μg sufentanil+10mg tropisetron (Luoxin Pharmaceutical, Approval No. H20061061), diluted with physiological saline to 100mL, load dose 0.06μg/kg. The occurrence of adverse reactions such as nausea and vomiting 24 hours after LC was recorded.

### Statistical Analysis:

SPSS 22.0 (IBM, Armonk, NY, USA) was used to analyze the data. The counting data were expressed as cases (%), and the differences between groups were compared by Chi-square test. The normality of the measurement data was verified by Shaprio-Wilk test. The normal distribution was expressed by the mean ± standard deviation, and t-test was used to compare the differences between groups. Repeated measures ANOVA was used to compare the inter-group and time effects. If it did not conform, it was expressed as median and interquartile range (Q25,Q75), and the non-parametric Mann-Whitney U rank sum test was performed. GraphPad Prism 8.0 software (GraphPad Software, San Diego, California, USA)) was used for drawing analysis. Bilateral α= 0.05 is the inspection level.

## RESULTS

A total of 113 patients were retrospectively enrolled in the study. Of them, 56 patients (24 males and 32 females with a mean age of 22.43±4.43 years) received ESP nerve block. There were 14 patients with ASA Grade-I and 42 patients with ASA Grade-II, and the mean operation time of the patients was 1.29 (SD of 0.37) hours. A total of 57 patients (26 males and 31 females with a mean age of 23.44±3.47 years) received TAP nerve block. There were 20 patients with ASA Grade-I and 37 patients with ASA Grade-II, and the mean operation time was 1.39 (SD of 0.40) hours. There were no differences in age, sex, ASA and operation time between the two groups (*P*>0.05) (Table-I).

**Table-I:** Comparison of baseline data of two groups of patients.

Group	n	Age (years)	Gender (n) M/F	BMI (kg/m^2^)	ASA Grade-I/II	Operation time (hours)
ESP-group	56	51.34±11.09	24/32	22.43±4.43	14/42	1.29±0.37
TAP-group	57	52.30±12.38	26/31	23.44±3.47	20/37	1.39±0.40
*t/χ^2^*	-	-0.433	0.087	-1.342	1.367	-1.337
*P*	-	0.666	0.768	0.182	0.242	0.184

**Fig.1:**
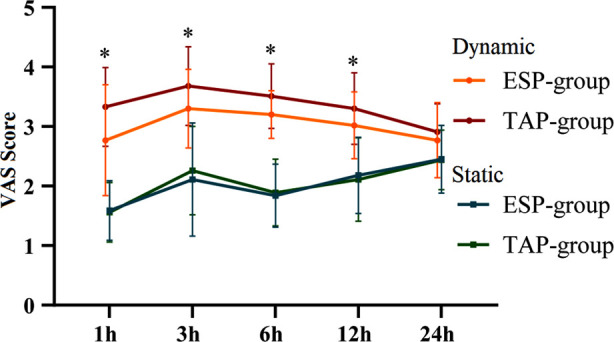
Comparison of VAS scores between the two groups in static and dynamic state.

We compared static VAS (sVAS) score of the two groups at each time node after the operation, and found that both TAP and ESP have similar static VAS scores (*P*>0.05). However, for the dynamic VAS (dVAS), there was a significant difference between the two groups at one hour, three hour, six hours and 12 hours after operation (*P*<0.05). The VAS scores at one hour, three hour, six hours and 12 hours after operation in the ESP-group were lower than those in the TAP-group (*P*<0.05). There was no difference in VAS score 24 hours after the operation (*P*>0.05) (Table-II, Fig.1).

**Table-II:** Changes of VAS score of subjective pain perception in two groups after operation.

VAS score	Time node	ESP-group (n=56)	TAP-group (n=57)	t	P
Static	One hour after operation	1.59±0.50	1.56±0.50	0.297	0.767
	Three hours after operation	2.11±0.95	2.26±0.74	-0.972	0.333
	Six hours after operation	1.84±0.53	1.89±0.56	-0.541	0.589
	12-hour after operation	2.18±0.64	2.11±0.70	0.583	0.561
	24-hour after operation	2.45±0.57	2.44±0.50	0.078	0.938
F		F_time_=43.226, F_between group_=0.069, F_interaction_=0.776		-	-
P		P_time_<0.001, P_between group_=0.793, P_interaction_=0.514		-	-
Dynamic	One hour after operation	2.77±0.93	3.33±0.66	-3.715	0.000
	Three hour after operation	3.3±0.66	3.68±0.66	-3.071	0.003
	Six hour after operation	3.2±0.4	3.51±0.54	-3.492	0.001
	12-hour after operation	3.02±0.56	3.30±0.6	-2.584	0.011
	24-hour after operation	2.77±0.63	2.91±0.47	-1.376	0.172
F		F_time_=27.526, F_between group_=18.061, F_interaction_=2.504		-	-
P		P_time_<0.001, P_between group_<0.001, P_interaction_=0.055		-	-

**Fig.2:**
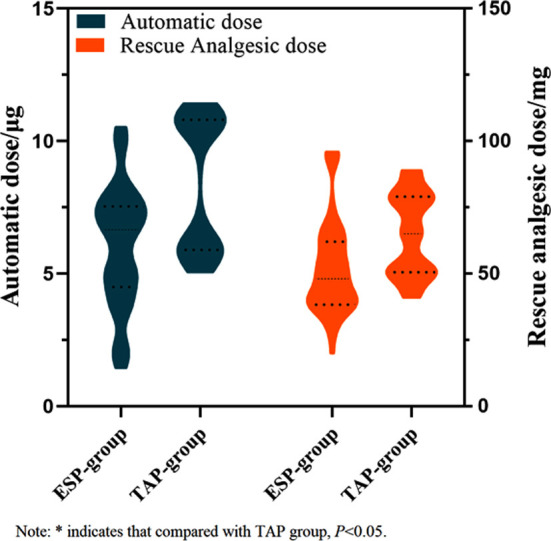
Comparison of patient-controlled dose and remedial analgesia dose between the two groups.

In terms of analgesia requirements, the patient-controlled dose and the remedial analgesia dose of the ESP-group were significantly lower than those of the TAP-group (*P*<0.05) (Table-III).

**Table-III:** Comparison of patient-controlled dose and remedial analgesic dose between the two groups.

Group	n	Patient-controlled dose (μg)	Remedial analgesic dose (mg)
ESP-group	56	6.65(4.61,7.53)	48(38.5,62)
TAP-group	57	8.99(5.91,10.74)	65(51,78)
Z	-	-4.003	-4.035
P	-	<0.001	<0.001

There were 12 reported cases of postoperative nausea and vomiting. Of them, five cases were in the ESP-group, with an incidence of 8.93% (5/56). There were seven cases of reported adverse effects in the TAP-group, with an incidence of 12.28% (7/57). There was no significant difference in the incidence of postoperative nausea and vomiting between the two groups (correction *χ^2^*=0.575, *P*=0.448). No obvious additional adverse reactions such as skin itching and excessive sedation were reported in both groups.

## DISCUSSION

The results of this study showed that both ESP block and TAP block have achieved good analgesic effect in patients undergoing LC. Liheng L *et al* 13 carried out a systematic review study that included 10 randomized controlled trials (RCT) of 570 patients and showed that ESP significantly reduced the consumption of opioids in the first 24 hours after surgery and improved the pain score compared with TAP. ESP also prolonged the anesthesia time and reduced postoperative nausea and vomiting. Altıparmak B *et al*^6^ carried out a RCT and showed that ultrasound-guided ESP block was more effective than TAP block in reducing postoperative tramadol consumption and pain score after LC. The above results are consistent with our study. Generally, T6~L1 nerve branches are blocked, and the range of sensory nerve block may vary depending on the blocking approaches.^6^,^13^ TAP block through subcostal approach can act on T6~T10 segments, and therefore is more suitable for upper abdominal surgery.^14^ ESP block can be quickly and accurately performed with low technical difficulty and have little impact on respiratory and circulatory system.^13^,^14^

In this study, the dynamic VAS score of the ESP-group was lower than that of the TAP-group at one hour, three hours, six hours and 12 hours after the operation. At the same time, the dose of patient-controlled medication and the dose of remedial analgesia in the ESP-group were lower than that of the TAP-group, suggesting that compared with the TAP nerve block, the application of preoperative ESP nerve block during LC surgery can reduce the dynamic pain score of patients at the early stage after the operation and significantly reduce the dose of opioids and other analgesics used after the operation. Presumably, the reason for this is that ESP nerve block is more efficient in promoting the skin diffusion of local anesthetic drugs on the fascia plane compared to the TAP nerve block. Boules ML *et al*^15^ compared the analgesic effects of the ESP and the TAP blocks during cesarean section. The results showed that compared with TAP, ESP block provides more effective pain relief, has a longer analgesic effect, and is associated with reduced use of analgesic and opioid drugs. While subcostal TAP block can effectively relieve postoperative pain and significantly improve the patient’s respiratory function, the diffusion direction in the transverse plane of the abdomen is mainly forward, but less backward.^16^

Other studies have also found that the analgesic effect of TAP block via subcostal approach is mainly reflected in the whole anterior abdominal wall, while the effect of lateral abdominal wall and posterior abdominal wall is very small.^17^ Therefore, when TAP block under ultrasound guidance is applied to LC, the patient’s side abdominal wall and rear abdominal wall may have discomfort, which may lead to more obvious pain sensation after the operation.^15^ TAP block has a good blocking effect on body pain after LC, but it has no obvious effect on visceral pain, resulting in insufficient analgesia. As a result, the dynamic pain feeling of LC patients who received TAP block is more significant than that of the patients who received the ESP block.^6^

On the other hand, ESP method blocks not only the somatic but also the visceral nerve fibers. Therefore, the degree of postoperative pain in patients is lower, making this method relatively more suitable for abdominal surgery.^18^,^19^ ESP block affects lower thoracic level, and the local anesthetic can diffuse, penetrate, and gradually extend into the thoracic paravertebral space.^15^ From this point of view, ESP blocking can also have an effect on the communicating branch fibers, which blocks the signal transmission pathway of sympathetic ganglia.^20^

In addition, no difference was found in the incidence of postoperative nausea and vomiting between the ESP-group and the TAP-group, suggesting that both methods were safe and reliable. However, some studies have shown that the incidence of postoperative nausea and vomiting in patients with ESP block is lower than that of the TAP block.^21^,^22^ ESP block has a wide range of clinical applications, and can be used for perioperative analgesia in different surgical operations such as spine, breast and knee joint surgeries.^6^,^13^ Furthermore, previous studies have shown that the main methods of blocking the somatic and visceral nerves include epidural analgesia, lumbar quadratus muscle block, and lumbar paravertebral block. However, these pain relief methods are more complex, time-consuming, and prone to serious complications.^6^,^15^

### Limitation:

It is a single-center study with a small sample size and short-term follow-up. Furthermore, only nausea and vomiting were studied, other potential postoperative adverse events were not studied. Further multi-center, large-sample prospective studies are needed to verify our findings.

## CONCLUSION

In patients undergoing LC, preoperative ultrasound-guided ESP block can significantly relieve the perioperative pain symptoms compared with TAP block, and can reduce the dose of perioperative opioids and remedial analgesic drugs. ESP block is safe, reliable and easy to administer.

### Authors’ contributions:

**HC** & **JL:** Conceived and designed the study.

**JZ** and **XZ:** collected the data and performed the analysis.

**HC** and **JL:** were involved in the writing of the manuscript and is responsible for the integrity of the study.

All authors have read and approved the final manuscript.
